# Deciphering the miRNA–TF–mRNA Regulatory Network Underlying Oocyte Maturation in Orange-Spotted Grouper (*Epinephelus coioides*): Insights from Oocyte mRNA-Seq and miRNA-Seq

**DOI:** 10.3390/ani16101549

**Published:** 2026-05-19

**Authors:** Mingqing Zhang, Yuting Wang, Dejin Liang, Donglan Diao, Meifang Li, Yingshi Tang, Yonglin Miao, Yuqing Yang, Su Liu, Jinhui Wu, Yong Zhang, Shuisheng Li

**Affiliations:** 1 State Key Laboratory of Biocontrol, School of Life Sciences, Southern Marine Science and Engineering Guangdong Laboratory (Zhuhai), Guangdong Provincial Key Laboratory for Aquatic Economic Animals, Guangdong Provincial Engineering Technology Research Center for Healthy Breeding of Important Economic Fish, Sun Yat-sen University, Guangzhou 510275, China; 2 Agro-Tech Extension Center of Guangdong Province, Guangzhou 510275, China

**Keywords:** *Epinephelus coioides*, oocyte, hydration, high-throughput sequencing

## Abstract

Oocyte maturation is a pivotal event in teleost reproduction that directly determines egg quality, fertilization success, and the developmental competence of early embryos. However, the transcriptional and post-transcriptional regulatory mechanisms operating within oocytes during maturation in marine teleosts remain poorly understood. In the present study, the orange-spotted grouper (*Epinephelus coioides*), an economically important marine aquaculture species, was used as a model. Oocytes at four distinct maturation stages were obtained by microscopically removing the surrounding follicular layers, followed by integrated mRNA-seq and miRNA-seq analyses to characterize the molecular regulatory landscape underlying oocyte maturation and hydration. The results showed that, as maturation progressed, oocyte diameter and wet weight increased significantly, accompanied by a marked decrease in Na^+^ content, a significant increase in K^+^ content, and the continuous accumulation of most free amino acids, indicating the gradual establishment of an osmotic basis favorable for oocyte hydration. Transcriptomic analysis further revealed extensive transcriptional remodeling during both the early and late phases of maturation. Differentially expressed genes were significantly enriched in pathways related to oocyte meiosis, cytokine signaling, lipid metabolism, DNA replication, cell cycle regulation, ribosome biogenesis, spliceosome function, oxidative phosphorylation, and mitochondrial activity, suggesting that oocyte maturation is a dynamic process characterized by a shift from basal growth maintenance to metabolic reprogramming, maternal transcript remodeling, and terminal maturation responses. miRNA profiling identified a large number of stage-specific differentially expressed miRNAs, including let-7d-5p, miR-22a-3p, and novel-miR-20/27/118, whose predicted target genes were mainly enriched in ribosome-related pathways, oxidative phosphorylation, DNA replication, transcriptional regulation, and signal transduction. Moreover, the miRNA–TF–mRNA regulatory network demonstrated that miRNAs may not only directly repress target genes, but also mediate hierarchical regulatory cascades through transcription factors, thereby coordinately participating in cell cycle progression, cytoskeletal remodeling, vesicular transport, and immune- and cell communication-related responses. Collectively, this study provides the first systematic temporal atlas of mRNA and miRNA regulation during oocyte maturation and hydration at the oocyte level in a marine teleost, thereby deepening our understanding of the molecular basis of meiotic resumption and egg quality formation, and offering valuable theoretical support for the optimization of artificial breeding and the identification of key molecular targets in grouper reproduction.

## Simple Summary

Successful egg development is essential for fish reproduction and for the stable production of high-quality fries in aquaculture. However, the internal molecular changes that take place inside an egg cell as it mature are still not well understood in marine fish. In this study, we used the orange-spotted grouper, an important farmed marine fish, to examine egg cells at four stages of maturation. By analyzing changes in gene activity and small regulatory molecules that control genes, we found that maturing egg cells gradually built up the conditions needed for water uptake, including changes in ions and free amino acids. At the same time, the egg cells showed major shifts in energy production, cell division, and the storage and use of maternal information needed for later development. We also identified key regulatory molecules that may help coordinate these changes. This study provides the first detailed timeline of molecular regulation during egg maturation in an egg cell of a marine fish. These findings improve our understanding of how good-quality eggs are formed and may help support better breeding strategies in aquaculture.

## 1. Introduction

In addition to the nutrition and genetics of the breeding stock, oocyte maturation is one of the most critical physiological processes in the reproduction of teleosts, as it directly determines egg quality, fertilization success, and the developmental competence of early embryos [1,2]. In marine aquaculture species, the acquisition of high-quality mature oocytes is essential not only for the success of artificial breeding, but also for the preservation of superior germplasm resources and the sustainable supply of commercial seedlings [3,4]. Oocyte maturation in teleosts is governed by a highly conserved and precisely coordinated hypothalamic–pituitary–gonadal (HPG) axis [4,5]. Specifically, when oocytes enter the maturational phase, the preovulatory surge of luteinizing hormone (LH) acts on the surrounding follicular layers, including granulosa cells and theca cells, thereby stimulating the synthesis and secretion of maturation-inducing steroids (MIS), such as 17α,20β-dihydroxy-4-pregnen-3-one (17,20β-DHP) [5,6]. MIS subsequently binds to specific membrane progestin receptors (mPRs) located on the oocyte membrane, activating non-genomic signaling pathways, reducing intracellular cyclic adenosine monophosphate (cAMP) levels, and ultimately promoting the activation of the cyclin B/CDK1 complex, also known as maturation-promoting factor (MPF) [5,7,8]. Once activated, MPF directly drives the resumption of meiosis from meiotic arrest and facilitates the progression of oocytes into the maturational and preovulatory stages, leading to germinal vesicle breakdown (GVBD) [5,9]. Taken together, oocyte maturation is not a single hormone-triggered event, but rather a tightly coordinated process involving endocrine signaling, membrane receptor-mediated responses, and intracellular cAMP-associated regulatory networks.

Compared with many freshwater or demersal-spawning fishes, one of the most distinctive features of oocyte maturation in marine teleosts is the occurrence of pronounced hydration concomitant with meiotic resumption [10,11]. This process enables oocytes to rapidly absorb water and increase in volume, thereby providing the physical and physiological basis for buoyancy maintenance, osmotic homeostasis, and early embryonic survival in the hyperosmotic marine environment [10]. Oocyte hydration is accompanied by a highly complex osmotic reorganization [12]. Large amounts of inorganic ions, such as K^+^ and Na^+^, are redistributed through membrane transport systems, while vitellogenin (Vtg), which accumulates in the growing oocyte during vitellogenesis, is hydrolyzed by cathepsins to generate abundant free amino acids (FAAs) [12]. These FAAs, together with inorganic ions, serve as major osmotic effectors and establish a strong intracellular osmotic gradient [13,14]. Driven by this gradient, aquaporins, particularly the teleost-specific Aqp1ab, are trafficked to the oocyte plasma membrane, where they mediate the rapid and massive influx of water into the oocyte, resulting in marked cell swelling and cytoplasmic clarification [15,16]. Therefore, oocyte maturation in marine teleosts should not be regarded merely as the resumption of meiosis, but rather as a complex physiological process integrating solute transport, ionic homeostasis, and yolk proteolysis/remodeling.

Although substantial progress has been made in understanding HPG axis-mediated endocrine regulation and oocyte hydration, our knowledge of the mechanisms governing oocyte maturation in marine teleosts, particularly in economically important marine aquaculture species, remains incomplete. In particular, post-transcriptional regulation and the dynamic changes in maternal transcripts within the oocyte during maturation are still poorly understood. In recent years, high-throughput sequencing technologies, including mRNA-seq and miRNA-seq, have been widely applied in the reproductive biology of aquaculture fishes [17,18]. Li et al. [19] in the study of gonadal microRNAs in *Acanthopagrus latus*, identified members of the let-7 and miR-200 families as key regulators involved in gametogenesis and sex differentiation. More recently, Li et al. [20] combined mRNA and miRNA sequencing to elucidate the mechanisms underlying impaired gonadal development in hybrid yellow catfish (*Pelteobagrus fulvidraco* ♀ × *P. vachelli* ♂), demonstrating that miRNAs such as ipu-miR-194a and ipu-miR-499 contribute to sterility by targeting genes associated with spermatogenesis. Collectively, these studies highlight the utility of integrated mRNA and miRNA sequencing for identifying key molecular targets involved in fish germ cell development. However, most of these investigations have focused on ovarian tissue or on the follicle complex containing both the oocyte and surrounding somatic cells, rather than directly addressing the transcriptional and post-transcriptional regulatory landscape of individual oocytes during maturation [18,21,22].

To address this gap, the present study used the orange-spotted grouper (*Epinephelus coioides*), a representative marine aquaculture species producing pelagic eggs, as the experimental model. By precisely removing the follicular layers through micromanipulation, we successfully isolated oocytes. Through integrated mRNA-seq and miRNA-seq analyses of oocytes collected at different maturational stages, we, for the first time, generated a comprehensive intracellular landscape of mRNA and miRNA dynamics during oocyte maturation and hydration in *E. coioides*. This study not only fills an important gap in our understanding of maternal miRNA–mRNA regulatory networks at the oocyte level in marine teleosts, but also provides new insights into the molecular mechanisms underlying meiotic resumption, hydration, and ovulation from both transcriptional and post-transcriptional perspectives. Moreover, our findings provide a theoretical foundation and valuable molecular resources for optimizing artificial breeding techniques in groupers and for improving the theoretical framework of reproductive regulatory biology in marine fishes.

## 2. Material and Methods

### 2.1. Ethical Statement

All animal experiments were conducted in accordance with the guidelines and were approved by the Animal Research and Ethics Committee of Sun Yat-sen University.

### 2.2. Sample Collection

**Figure 1:**
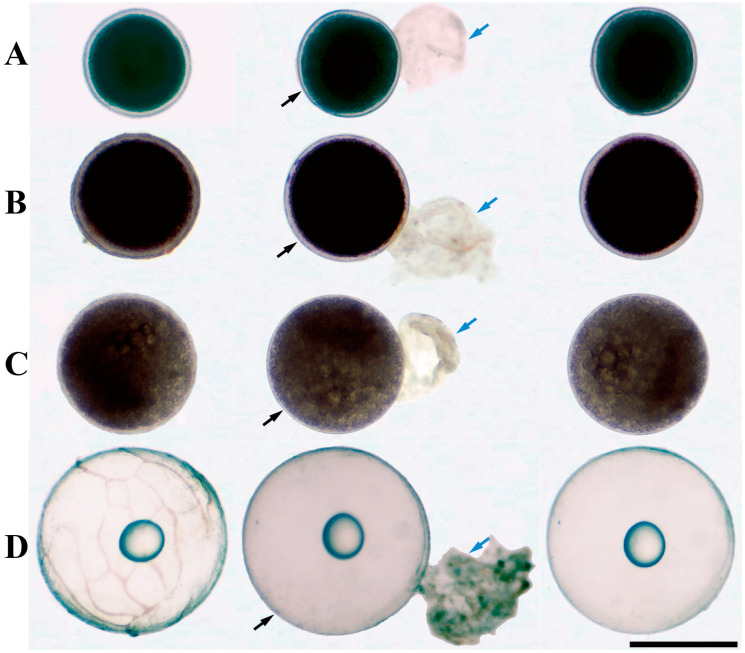
Morphological changes during oocyte maturation. (**A**) S1; (**B**) S3; (**C**) S5; (**D**) S8. Black arrows: oocytes; blue arrows: follicular layer. Scale bars = 500 μm.

Broodstock of *E. coioides* used in this study were maintained at the Guangdong Marine Fisheries Experimental Center (Huizhou, China). During the reproductive season from April to May 2024, appropriate amounts of ovarian tissue were collected by gentle aspiration using a sterile plastic catheter for the isolation of follicles at different maturational stages, and the follicular layers were subsequently removed with fine forceps to obtain oocytes. Briefly, the aspirated ovarian tissue was transferred into Leibovitz’s L-15 medium (without phenol red, supplemented with 1% penicillin–streptomycin–amphotericin B; Procell, Wuhan, China). Under a stereomicroscope (SMZ-168, Motic, Xiamen, China), follicles representing four maturational stages were identified and selected according to our previous study (Figure 1) [11,23]. Follicles were manually separated with fine forceps, and approximately 200 follicles were collected for each stage. The follicular layers were then carefully stripped away from follicles at each maturational stage to obtain oocytes. These oocytes were collected into sterile 1.5-mL microcentrifuge tubes, washed three times with 1 × PBS (Gibco, Shanghai, China) to remove residual L-15 medium, and immediately frozen in liquid nitrogen for subsequent transcriptomic sequencing. Three biological replicates were prepared for each stage.

In addition, to assess changes in dry weight, wet weight, Na^+^ and K^+^ ion contents, and amino acid levels during oocyte maturation, 100 oocytes from each maturational stage were isolated and transferred into sterile 1.5-mL microcentrifuge tubes. The oocytes were washed three times with 1 × PBS to remove residual L-15 medium, and excess PBS was carefully removed with absorbent paper as completely as possible. Samples were then rapidly frozen in liquid nitrogen and stored for subsequent analyses. Three biological replicates were included for each stage.

### 2.3. Determination of Oocyte Dry Weight, Wet Weight, Na^+^ and K^+^ Contents, and Amino Acid Levels

An analytical balance (0.0001 g, YINGHENG, Hangzhou, China) was used to determine the mass of each microcentrifuge tube for the calculation of oocyte wet and dry weights. Briefly, the mass of the microcentrifuge tube containing fresh oocytes was first recorded. The tube was then dried in a constant temperature oven (DHG-101, SUBO, Hangzhou, China) at 60 °C to a constant weight, after which its mass was measured again. Each tube was weighed three times, and the mean value was used for subsequent calculations. Oocyte dry and wet weights were calculated according to the following formula:

Oocyte weight (μg/oocyte) = (mass of tube containing oocytes − mass of empty tube)/100

The Na^+^ and K^+^ contents, as well as amino acid levels, were determined by Guangzhou Gene Denovo Biotechnology Co., Ltd. (Guangzhou, China). The Na^+^ and K^+^ were quantified using microwave digestion coupled with inductively coupled plasma mass spectrometry (ICP-MS) [24], whereas amino acid contents were analyzed using liquid chromatography–tandem mass spectrometry (LC-MS/MS) [25].

### 2.4. Library Construction and Sequencing

Total RNA was extracted from all oocyte samples using TRIzol reagent (Invitrogen, Waltham, MA, USA). RNA quality and integrity were assessed using an Agilent 2100 Bioanalyzer (Agilent Technologies, Palo Alto, CA, USA) and 1% agarose gel electrophoresis. A total of 12 high-quality RNA samples, characterized by three distinct bands (5S, 18S, and 28S rRNA) and OD260/280 ratios ranging from 1.8 to 2.2, were selected for library construction and sequencing.

For mRNA sequencing, cDNA libraries were constructed using the NEBNext^®^ Ultra™ RNA Library Prep Kit (NEB #E7530, New England Biolabs, Ipswich, MA, USA). Briefly, mRNA was enriched using Oligo(dT) magnetic beads and subsequently fragmented using fragmentation buffer. The fragmented mRNA was reverse-transcribed into cDNA, followed by second-strand synthesis. The resulting double-stranded cDNA fragments were end-repaired, A-tailed, and ligated to Illumina sequencing adapters. The ligation products were purified using AMPure XP beads (1.0×), size-selected by agarose gel electrophoresis, and amplified by PCR to generate the final cDNA libraries. Library quality was evaluated using the DNA 1000 Kit (Agilent Technologies, Palo Alto, CA, USA). The libraries were sequenced on an Illumina NovaSeq 6000 platform at Gene Denovo Biotechnology Co., Ltd. (Guangzhou, China). Raw sequencing data for mRNA have been deposited in the CNCB database under accession number PRJCA061616.

For miRNA sequencing, the same RNA samples were used to construct small RNA libraries with the TruSeq Small RNA Sample Prep Kit (Illumina, San Diego, CA, USA), following the manufacturer’s instructions. Briefly, 3′ and 5′ adapters were sequentially ligated to the ends of small RNAs, followed by first-strand cDNA synthesis and PCR amplification. PCR products ranging from 140–160 bp were purified using 6% polyacrylamide Tris-borate-EDTA (TBE) gel electrophoresis to obtain the final miRNA libraries. Sequencing was performed on an Illumina HiSeq 2500 platform, generating 50 bp single-end reads. Raw sequencing data for miRNA have been deposited in the CNCB database under accession number PRJCA061597.

### 2.5. Identification of Differentially Expressed Genes (DEGs) and Functional Analysis

Raw sequencing reads were processed using fastp (v0.23.1) to remove adapter sequences and low-quality reads [26]. Clean reads were aligned to the reference genome of orange-spotted grouper (NCBI accession: GCA_051314025.1) using HISAT2 (v2.2.1) [27]. Gene expression levels were quantified at the gene level using StringTie (v2.2.1) and normalized as transcripts per million (TPM) [28]. Principal component analysis (PCA) and sample correlation heatmaps were generated using the PCAtools and pheatmap packages, respectively, to evaluate sample reproducibility and clustering patterns.

The DEGs between developmental stages were identified using DESeq2 (v1.34.0) [29]. Genes with |log_2_(fold change)| > 1 and *p* < 0.05 were considered significantly differentially expressed. Gene Ontology (GO) and Kyoto Encyclopedia of Genes and Genomes (KEGG) enrichment analyses were performed using the clusterProfiler package (v4.2.2). Functional annotations were obtained from the EggNOG database using eggNOG-mapper (v2.1.6), and GO terms and KEGG pathways with *p* < 0.05 were considered significantly enriched [30]. To characterize the temporal expression patterns of DEGs during oocyte maturation, all DEGs were subjected to clustering analysis using the ClusterGVis package (v0.99.9). GO and KEGG enrichment analyses were further conducted for DEGs within each cluster.

### 2.6. miRNA Identification and Differential Expression Analysis

Raw sequencing data were subjected to stringent filtering to obtain high-quality clean reads. Reads that did not meet quality control criteria were removed, including those with a quality score < 20, containing ambiguous bases (N), lacking a 3′ adapter, containing a 5′ adapter, possessing poly-A tails, shorter than 18 nt, or with read counts < 2 [31]. Based on length distribution analysis, reads ranging from 18 to 35 nt were retained for subsequent analyses.

Clean reads were aligned to the miRBase database (v22) and Rfam database (v12.0) using Bowtie2 (v2.2.2) to identify and annotate known miRNAs, as well as other non-coding RNAs, including rRNA, tRNA, snRNA, and snoRNA. For unannotated clean reads, novel miRNAs were predicted by integrating mirvo (v1.3) and miRDeep (v2.0), based on the identification of Dicer cleavage sites, secondary structure characteristics, and minimum free energy of the candidate sequences [32].

The expression levels of all miRNAs (including known and novel miRNAs) were normalized across samples using the transcripts per million (TPM) method to generate a relative expression matrix. The normalization formula was as follows: TPM = (raw miRNA counts/total clean reads) × 10^6^ [33]. The PCA and sample correlation analyses were performed using the PCAtools and pheatmap packages, respectively. Differential expression analysis between groups was conducted using the edgeR package. miRNAs with |log_2_(fold change)| > 1 and *p* < 0.05 were defined as differentially expressed miRNAs (DE miRNAs).

### 2.7. miRNA Target Gene Prediction, Enrichment Analysis, and TF Identification

miRNA target genes were predicted using three independent algorithms: RNAhybrid (v2.1.2), miRanda (v3.3a), and TargetScan (v7.0). The intersection of the predicted targets from these three methods were defined as the final set of candidate target genes [34]. GO and KEGG enrichment analyses were subsequently performed on these target genes.

To identify TFs, DEGs obtained from transcriptome sequencing were aligned against the Animal Transcription Factor Database (Animal TFDB) using BLASTp v2.17.0 (http://www.ncbi.nlm.nih.gov/BLAST/) accessed on 23 January 2026 [35]. Identified TFs were further classified and quantified according to their respective families. Differential expression analysis of TFs between groups was performed using DESeq2, and differentially expressed TFs (DE TFs) were identified.

### 2.8. Construction of the miRNA-TF-mRNA Network

Based on the identified DE miRNAs, DEGs, and DE TFs, potential regulatory interactions were screened using Pearson correlation analysis. miRNA–mRNA and miRNA–TF pairs were selected under the criteria of Pearson correlation coefficient (PCC) < −0.7 and *p* < 0.05, while TF–mRNA pairs were filtered using |PCC| > 0.9 and *p* < 0.05. These interactions were integrated to construct a putative miRNA–TF–mRNA regulatory network, which was visualized using Cytoscape software (v3.6.0) [36].

### 2.9. Quantitative Real-Time PCR (qRT-PCR) Validation

Six DE miRNAs and six DEGs were selected for RT-qPCR validation. Specific primers for the DE miRNAs and DEGs were designed using Primer Premier 5.0, with *β-actin* and U6 was used as the reference genes for mRNA and miRNA, respectively (Table S1). Total RNA was extracted from 12 oocyte samples using TRIZOL reagent (Invitrogen, Waltham, MA, USA). cDNA synthesis for miRNA and gene was performed using the miRNA First Strand cDNA Synthesis Kit (Tailing Reaction) (Sangon Biotech, Shanghai, China) and the One-step gDNA Removal Kit (TransGen Biotech, Beijing, China), respectively. qRT-PCR reactions were prepared using the microRNAs qPCR Kit (SYBR Green Method) (Sangon Biotech, Shanghai, China) for miRNAs and the PerfectStart^®^ Green qPCR SuperMix (TransGen Biotech, Beijing, China) for DEGs. Each reaction mixture contained 8 μL RNase-free H_2_O, 10 μL 2× SYBR mix, 1 μL cDNA, and 0.5 μL each of forward and reverse primers. All experiments were performed with at least three independent biological replicates, and each assay included a no-template control. qRT-PCR was conducted on a LightCycler 480 system (Roche, Penzberg, Germany) under the following conditions: 95 °C for 30 s; followed by 40 cycles of 95 °C for 5 s, 60 °C for 15 s, and 72 °C for 10 s. Melting curve analysis was performed using default instrument settings to verify amplification specificity and detect primer-dimer formation. Relative expression levels of miRNAs and DEGs were calculated using the 2^−ΔΔCt^ method.

### 2.10. Statistical Analysis

All data are presented as the mean ± standard deviation (SD). Before statistical analysis, data were tested for normality and homogeneity of variance to ensure the assumptions of parametric tests were met. Normality was assessed using the Shapiro–Wilk test, and homogeneity of variance was evaluated using Levene’s test. Only datasets that satisfied of these assumptions were subjected to further analysis. Statistical analyses were performed using SPSS Statistics 25.0 (IBM, New York, NY, USA). Differences among groups were analyzed using one-way analysis of variance (ANOVA), followed by Tukey’s post hoc test for multiple comparisons when significant differences were detected. A significant level of *p* < 0.05 was applied throughout.

## 3. Results

### 3.1. Changes During Oocyte Maturation in E. coioides

Based on the staging criteria described by Tang et al. [11,23], four representative stages of oocyte maturation (26–28 °C) in *E. coioides* were selected for analysis: the fully grown stage (S1; before HCG injection; oocyte diameter 490–510 μm), characterized by completed vitellogenic accumulation, distinct yolk granules, and a centrally located nucleus; the S3 stage (20 h after HCG injection; 565–610 μm), in which oocyte diameter had increased markedly but no obvious yolk hydrolysis was observed; the S5 stage (24 h after HCG injection; 640–660 μm), characterized by the onset of yolk hydrolysis, and the appearance of oil droplets of variable sizes; and the S8 stage (32 h after HCG injection; 835–900 μm), in which yolk hydrolysis was complete, only a single oil globule remained, and the cytoplasm appeared transparent (Figure 1).

In addition, following the initiation of oocyte maturation, both oocyte diameter and wet weight increased significantly, whereas Na^+^ and K^+^ concentrations showed significant decreases and increases, respectively (*p* < 0.05). In contrast, dry weight did not change significantly (*p* > 0.05; Figure S1). Free amino acid analysis further showed that total amino acid content increased markedly as maturation progressed. Among the 63 amino acids detected, 46 exhibited a continuous increase during oocyte maturation (Table S2), indicating that the oocytes had established the osmotic basis required for hydration.

### 3.2. mRNA-Seq Analysis of the Oocyte

#### 3.2.1. Overview of mRNA-Seq Results

To better characterize the dynamic changes in endogenous mRNAs during oocyte maturation, oocytes at four distinct maturational stages were isolated for transcriptomic analysis (Figure 1). Transcriptome sequencing of the 12 oocyte samples generated a total of 509,012,979 raw reads. After removal of low-quality reads, 35,460,008 to 48,295,748 clean reads were retained for each sample, with Q20 and Q30 values exceeding 98.33% and 94.90%, respectively. The average GC content across all samples was 45.77% (Table S3). The mapping rates of all samples to the reference genome (GCA_051314025.1) ranged from 90.76% to 92.70%. In addition, Pearson’s correlation coefficient heatmap and PCA showed high reproducibility among biological replicates within each group (Figure S2). Collectively, these results indicate that the transcriptomic data were of high quality and suitable for subsequent analyses.

#### 3.2.2. DEGs Analysis

**Figure 2:**
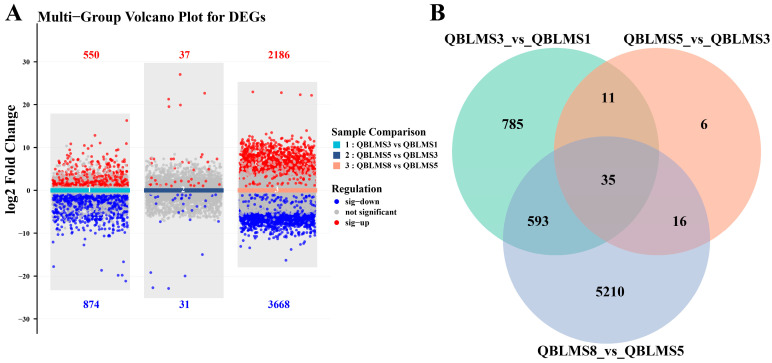
Venn diagram (**A**) and volcano plot (**B**) of DEGs across the three comparison groups during oocyte maturation in *E. coioides*.

Analysis of the three pairwise comparisons during oocyte maturation (QBLMS3 vs. QBLMS1, QBLMS5 vs. QBLMS3, and QBLMS8 vs. QBLMS5) revealed that extensive transcriptional regulation occurred during the early and late stages of maturation. Specifically, 550 and 2186 genes were significantly upregulated, whereas 874 and 3668 genes were significantly downregulated, in the early and late comparisons, respectively. In contrast, only 37 genes were significantly upregulated and 31 genes were significantly downregulated during the intermediate stage (Figure 2A). Integrated analysis of these DEGs showed that 35 genes were consistently differentially expressed throughout the entire maturation process (Figure 2B). Notably, the late maturation stage contained the largest number of stage-specific DEGs, reaching 5210, indicating that extensive transcriptional reprogramming occurs during final oocyte maturation and may play critical roles in the completion of GVBD and ovulation.

**Figure 3:**
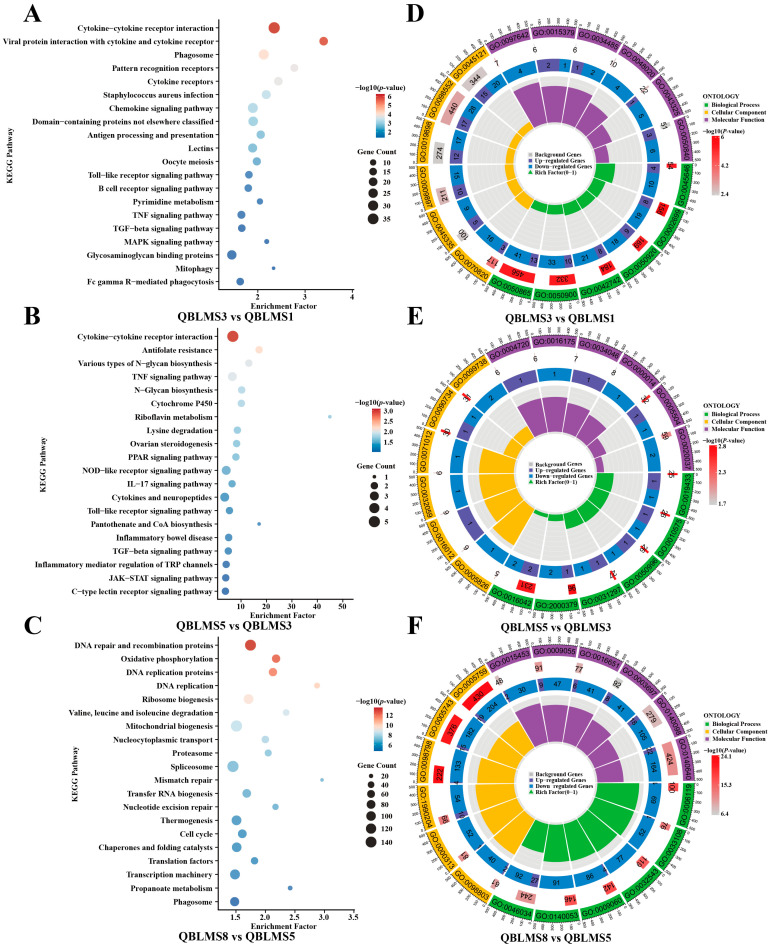
KEGG (**A**–**C**) and GO (**D**–**F**) enrichment analyses of DEGs during oocyte maturation in *E. coioides*.

KEGG enrichment analysis was performed on the DEGs identified from the three pairwise comparisons described above, resulting in the annotation of 377, 130, and 414 KEGG pathways, respectively. The top 20 significantly enriched pathways (*p* < 0.05) for each stage are presented in Figure 3A–C. The results showed that during the initiation of oocyte maturation (S1–S3), DEGs were mainly enriched in cytokine–cytokine receptor interaction, chemokine signaling pathway, pattern recognition receptor signaling, phagosome, antigen processing and presentation, and oocyte meiosis, suggesting that cell signaling, immune responses, and meiotic regulatory processes were already activated at the onset of maturation. During the mid-maturation stage (S3–S5), pathways such as cytokine–cytokine receptor interaction, TNF signaling pathway, ovarian steroidogenesis, cytochrome P450, PPAR signaling pathway, and N-glycan biosynthesis were significantly enriched, indicating that this stage was primarily characterized by endocrine metabolism and molecular modification. In the final stage of maturation (S5–S8), oocytes shifted toward extensive remodeling of fundamental cellular metabolism, with significant enrichment of pathways involved in DNA replication, DNA repair, cell cycle, ribosome biogenesis, spliceosome, translation factors, oxidative phosphorylation, and mitochondrial biogenesis, thereby supporting the extensive accumulation of maternal mRNAs and proteins.

Further GO enrichment analysis was performed on the identified DEGs, and the top six enriched GO terms in each category are shown in Figure 3D–F. The results revealed that during the initiation stage of oocyte maturation (S1–S3), DEGs were mainly enriched in terms related to regulation of immune effector process, regulation of chemotaxis, defense response, cell activation, as well as membrane raft, phagocytic vesicle, and chemokine receptor binding. These findings suggest the activation of membrane receptor-mediated sensing, extracellular signal responsiveness, and ion transport-related processes at the onset of maturation. During the mid-maturation stage (S3–S5), enrichment of GO terms such as triglyceride and lipid catabolic process, regulation of reactive oxygen species metabolic process, replication fork processing, site of DNA damage, and cell cortex region indicated that this stage was characterized primarily by lipid metabolic remodeling, redox regulation, and dynamic cytoskeletal reorganization. In the final stage of maturation (S5–S8), DEGs were significantly enriched in oxidative phosphorylation, aerobic respiration, mitochondrial translation, mitochondrial gene expression, ATP metabolic process, and electron transport, indicating a marked enhancement of mitochondrial energy metabolism and nucleic acid processing during late maturation. These temporally coordinated transcriptional dynamics not only ensure the proper resumption of meiosis and the precise synchronization of nuclear and cytoplasmic maturation, but also establish the essential material and energetic reserves required for subsequent fertilization and early embryonic development.

#### 3.2.3. Temporal Expression Pattern Analysis of DEGs

**Figure 4:**
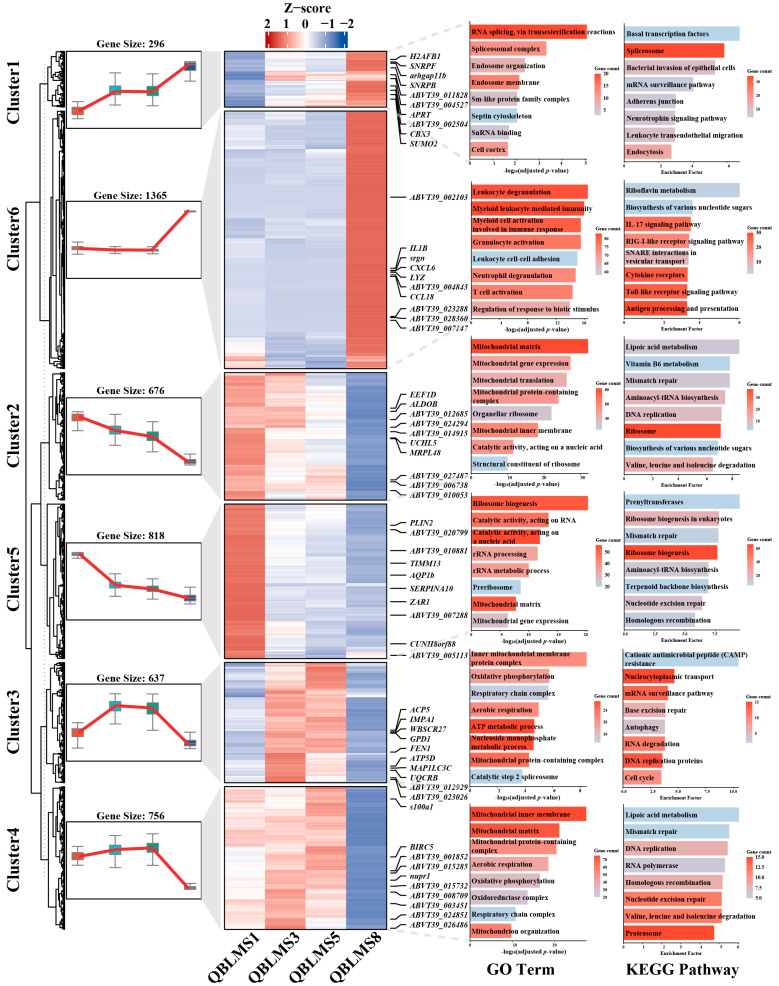
Expression trend analysis of DEGs during oocyte maturation in *E. coioides*. The left panel shows boxplots of gene expression trends, the middle panel presents the clustered heatmap of expression profiles with representative genes labeled for each cluster, and the right panel shows the GO and KEGG enrichment analyses for each cluster.

The temporal expression pattern analysis of the DEGs identified during oocyte maturation revealed six representative expression clusters (Figure 4). Overall, distinct clusters exhibited clear stage-specific dynamic patterns throughout maturation. Cluster 2 (676 genes) and Cluster 5 (818 genes) showed an overall downward trend. Cluster 2 gradually decreased from S1 to S8 and was mainly enriched in pathways and processes related to ribosome, DNA replication, aminoacyl-tRNA biosynthesis, and mitochondrial translation, with representative genes including *UCHL5*, *ALDOB*, *EEF1D*, and *MRPL48*. Cluster 5 declined more markedly from S1 to S3 and was primarily associated with ribosome biogenesis, nucleotide excision repair, homologous recombination, and rRNA processing, with representative genes including *PLIN2*, *ZAR1*, *AQP1b*, and *TIMM13*. These results suggest that, as oocytes transition from the growth phase to the maturational phase, processes involved in basal protein synthesis, ribosome biogenesis, and maintenance of early growth are progressively suppressed. In contrast, Cluster 1 (296 genes) displayed a continuous increase from S1 to S8 and was significantly enriched in basal transcription factors, spliceosome, mRNA surveillance pathway, ribonucleoprotein complex biogenesis, and mitochondrial translation. This cluster included key genes such as *H2AFB1*, *SUMO2*, *CBX3*, *SNRPB*, and *SNRPF*, indicating that basal transcriptional regulation, RNA processing and surveillance, and mitochondrial protein translation are progressively enhanced as maturation proceeds. Cluster 3 (637 genes) showed a transient increase from S1 to S3, followed by a gradual decline toward S8. It was mainly enriched in autophagy, RNA degradation, cell cycle, oxidative phosphorylation, and ATP metabolic process, and contained representative genes such as *s100a1*, *UQCRB*, *MAP1LC3C*, *ATP5D*, *FEN1*, and *GPD1*, suggesting a primary role in energy supply and metabolic regulation during early maturation. Cluster 4 (756 genes) increased slightly from S1 to S5 and then declined, with significant enrichment in DNA replication, RNA polymerase, proteasome, and mitochondrial organization, and representative genes including *BIRC5* and *nupr1*. Notably, Cluster 6 (1365 genes) showed relatively minor changes from S1 to S5 but increased sharply at S8. This cluster was mainly enriched in the IL-17 signaling pathway, RIG-I-like receptor signaling pathway, Toll-like receptor signaling pathway, antigen processing and presentation, RNA splicing, nuclear body organization, and spliceosomal complex. Key genes included *CCL18*, *LYZ*, *CXCL6*, *srgn*, and *IL1B*, indicating that the endogenous mRNA expression landscape undergoes substantial remodeling during final oocyte maturation, accompanied by enhanced RNA splicing, vesicular trafficking, and immune-related signaling activity. Collectively, these temporal changes in endogenous mRNA suggest a progressive shift during oocyte maturation from basal growth maintenance toward post-transcriptional regulation, metabolic remodeling, and terminal maturation-associated responses.

### 3.3. miRNA-Seq Analysis of the Oocyte

#### 3.3.1. Overview of miRNA-seq Results

A total of 9,508,277 to 17,203,366 raw reads were generated from miRNA sequencing of the 12 oocyte samples. After filtering out low-quality reads, 9,453,910 to 16,762,765 high-quality reads were retained for subsequent analyses (Table S4). The length distribution of these high-quality reads ranged from 18 to 35 nt, with 23-nt reads representing the most abundant class (Table S5). Reads of 20, 21, 22, and 23 nt accounted for relatively high proportions of the total high-quality reads, representing 10.19%, 11.19%, 12.80%, and 13.22%, respectively, indicating the high reliability of the sequencing data.

#### 3.3.2. Classification and Identification of Small RNAs

All clean reads were annotated and classified by alignment against the GenBank, Rfam, and miRBase databases to identify non-coding RNAs and known miRNAs (Table S6). Overall, rRNA, snRNA, snoRNA, and tRNA accounted for 25.07% to 75.20% of the clean reads. A total of 338,705 to 1,659,402 reads were identified as known miRNAs, representing 2.77% to 11.89% of the clean reads, indicating that only a relatively small proportion of the clean reads corresponded to miRNAs. Among the unannotated clean reads, 2446 to 17,132 were predicted as novel miRNAs.

#### 3.3.3. Differential Expression Analysis of miRNAs

The expression levels of both known and novel miRNAs were normalized using the TPM method to assess sample correlation and variability. PCA showed that PC1 and PC2 together explained 39.04% of the total variance, and the samples exhibited acceptable reproducibility, indicating that the dataset was suitable for subsequent analyses (Figure S3A). Venn diagram analysis showed that 214 miRNAs were shared among all groups, whereas QBLMmiS1 and QBLMmiS3 contained the largest numbers of uniquely DE miRNAs, with 13 and 17, respectively (Figure S3B).

**Figure 5:**
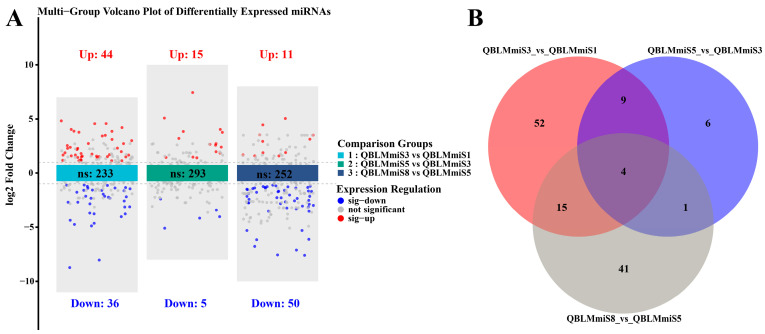
Volcano plot (**A**) and Venn diagram (**B**) of DE miRNAs across the three comparison groups during oocyte maturation in *E. coioides*.

Based on maturational stage, three pairwise comparisons of DE miRNAs were performed. As shown in Figure 5A, 80 DE miRNAs (44 upregulated and 36 downregulated), 20 DE miRNAs (15 upregulated and 5 downregulated), and 61 DE miRNAs (11 upregulated and 50 downregulated) were identified in the comparisons of QBLMmiS3 vs. QBLMmiS1, QBLMmiS5 vs. QBLMmiS3, and QBLMmiS8 vs. QBLMmiS5, respectively. Among these three comparisons, four DE miRNAs were shared across all groups. In addition, 52, 6, and 41 uniquely DE miRNAs were identified in QBLMmiS3 vs. QBLMmiS1, QBLMmiS5 vs. QBLMmiS3, and QBLMmiS8 vs. QBLMmiS5, respectively (Figure 5B).

#### 3.3.4. Target Prediction and Enrichment Analysis of DE miRNAs

**Figure 6:**
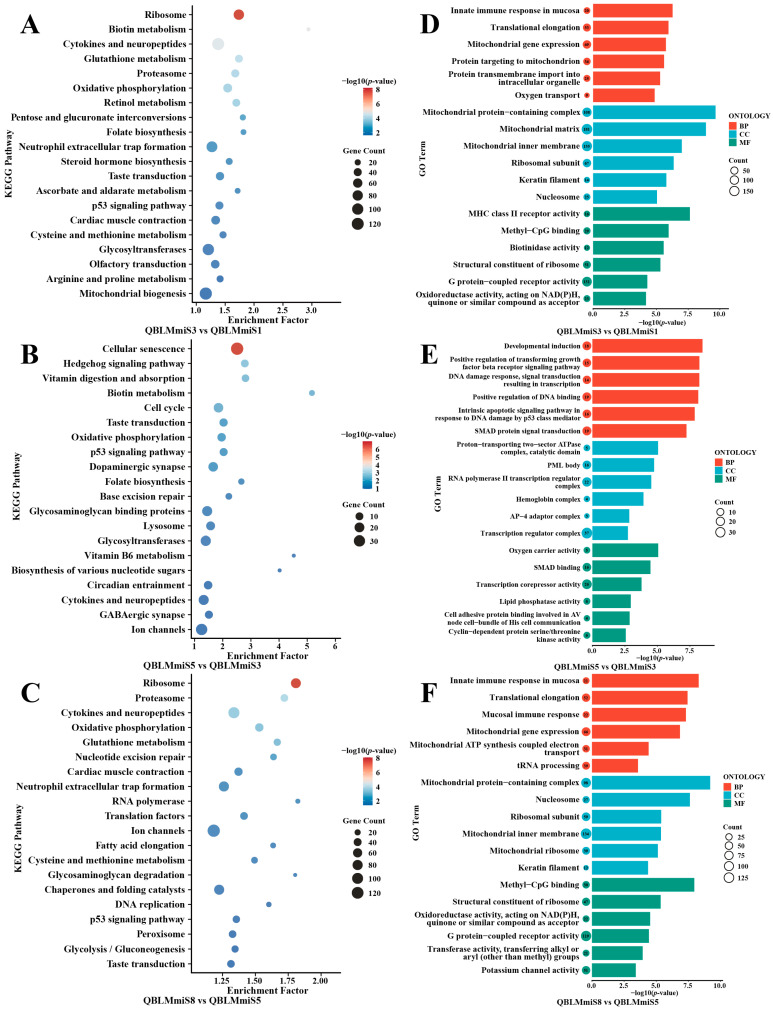
KEGG (**A**–**C**) and GO (**D**–**F**) enrichment analyses of target genes of DE miRNAs in *E. coioides*.

An overview of the predicted miRNA target genes in each sample is provided in Table S7. KEGG enrichment analysis showed that the significantly enriched pathways of target genes of DE miRNAs across the various stages of oocyte maturation were mainly associated with ribosome, cytokines and neuropeptides, oxidative phosphorylation, biotin metabolism, and the p53 signaling pathway. During late maturation, pathways related to DNA replication, RNA polymerase, transcription factors, and proteasome were also significantly enriched, suggesting that miRNA-mediated regulation is involved throughout protein synthesis and degradation, mitochondrial energy metabolism, redox homeostasis, and stress responses. GO enrichment analysis further revealed that these target genes were mainly enriched in terms related to mitochondrial gene expression, translational elongation, mitochondrial inner membrane, ribosomal subunit, oxidoreductase activity, and G protein-coupled receptor activity. During the mid-maturation stage, enriched terms also included TGF-β/SMAD signaling transduction, DNA damage response, and transcriptional regulation, indicating that DE miRNAs may participate in oocyte maturation by modulating mitochondrial function, translational activity, signal transduction, and nucleic acid metabolism (Figure 6).

### 3.4. Identification of TFs

A total of 947 TFs, belonging to 64 TF families, were predicted by mapping to the AnimalTFDB database. Among these, the zf-C2H2 family contained the largest number of TFs (257), followed by the MBD, Homeobox, and MYB families, which comprised 58, 55, and 48 TFs, respectively. Notably, 545 DE TFs were identified during the late stage of oocyte maturation (Figure S4A). In addition, 96 (36 upregulated and 60 downregulated), 3 (1 upregulated and 2 downregulated), and 624 (169 upregulated and 455 downregulated) DE TFs were detected in the comparisons of QBLMS3 vs. QBLMS1, QBLMS5 vs. QBLMS3, and QBLMS8 vs. QBLMS5, respectively (Figure S4B).

### 3.5. Construction of the miRNA–TF–mRNA Regulatory Network

**Figure 7:**
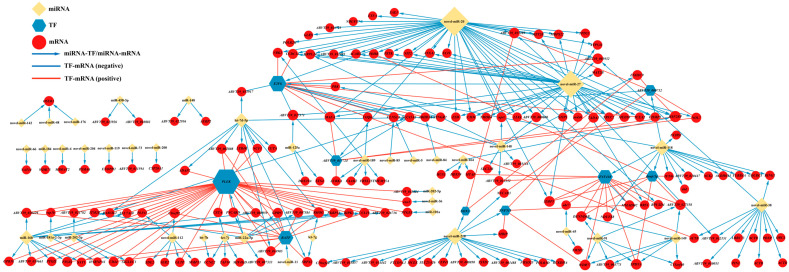
Putative miRNA–TF–mRNA regulatory network during oocyte maturation in *E. coioides*.

To further investigate the interactions and regulatory relationships among DE miRNAs, DE TFs, and DEGs during oocyte maturation, a putative miRNA–TF–mRNA regulatory network was constructed, comprising 211 miRNA–mRNA pairs, 12 miRNA–TF pairs, and 98 TF–mRNA pairs. As shown in Figure 7, this network revealed that miRNAs can not only directly repress the expression of their target genes, but also regulate specific TF, thereby forming complex indirect cascade modules and multilayered feedback networks. For example, let-7d-5p targeted *SYNGR2*, a gene associated with synaptic and vesicular transport (PCC = −0.915), as well as *SRPX2* (PCC = −0.875). Meanwhile, novel-miR-118 significantly downregulated the expression of the tubulin gene *TUBB4A* (PCC = −0.929), suggesting that these specific miRNAs may play central restrictive roles in maintaining oocyte microenvironmental homeostasis and cytoskeletal remodeling. More importantly, the network contained clear “miRNA–TF–mRNA” cascade regulatory axes. For instance, miR-22a-3p negatively targeted *PLEK* (PCC = −0.723), whereas *PLEK* showed highly significant positive correlations with a series of genes, including *CD44* (PCC = 0.977), *SIGLEC1* (PCC = 0.962), and *RIPK3* (PCC = 0.949). These results suggest that miR-22a-3p may indirectly suppress the expression of these cell communication- and response-related genes through inhibition of *PLEK*. Similarly, novel-miR-20 and novel-miR-27 jointly targeted *E2F6* (both PCC = −0.820), while *E2F6* in turn positively regulated genes closely associated with cell cycle progression and differentiation, such as *MAEA* (PCC = 0.958) and *VRK1* (PCC = 0.949). In addition, some core target genes were simultaneously subjected to post-transcriptional repression by miRNAs and transcriptional activation by TFs, thereby forming complex multilayered regulatory modules. For example, the transmembrane protein gene *ITM2B* and the G protein-coupled receptor gene *GPR97* were not only directly targeted by miR-22a-3p, but were also strongly correlated with *PLEK* (*PLEK*–*ITM2B*: PCC = 0.974; *PLEK*–*GPR97*: PCC = 0.948). Collectively, this regulatory network highlights the tight coupling between transcriptional and post-transcriptional regulation during oocyte maturation and provides an important framework for identifying key regulatory factors and elucidating their underlying molecular mechanisms.

### 3.6. RT-qPCR Validation

RT-qPCR validation showed that the expression patterns of the six DE miRNAs and six DEGs were generally consistent with those obtained from the sequencing data, thereby supporting the reliability and accuracy of the sequencing results (Figure S5).

## 4. Discussion

Using the orange-spotted grouper (*E. coioides*) as a model, the present study obtained oocytes at different maturational stages through micromanipulation and, for the first time, systematically characterized the dynamic changes in endogenous mRNAs and miRNAs during oocyte maturation and hydration in a marine teleost. By constructing a multilayered miRNA–TF–mRNA regulatory network, we demonstrated that oocyte maturation in *E. coioides* is not merely a process of meiotic resumption, but rather a continuous and coordinated event integrating osmotic regulation, maternal substance remodeling, RNA-mediated regulation, and terminal maturation-associated responses.

### 4.1. Molecular Basis of Hydration and Osmotic Regulation

One of the most prominent features of oocyte maturation in marine teleosts is the synchronous occurrence of meiotic resumption and rapid hydration [10,37]. In the present study, oocyte wet weight and diameter increased significantly as maturation progressed, accompanied by a marked decrease in Na^+^, a significant increase in K^+^, and continuous accumulation of FAAs. The sustained upregulation of *catb*, *catl*, and *catd* during maturation suggests that enhanced yolk proteolysis may represent an important source of FAAs accumulation, whereas the stage-specific expression changes in *nkatα1* and *nkatβ1* indicate that Na^+^,K^+^-ATPase-mediated ion transport plays a critical role in the initiation and maintenance of hydration [11,13]. These findings are highly consistent with the classical mechanism of oocyte hydration in marine teleosts. On this basis, the teleost-specific aquaporin Aqp1ab (formerly termed Aqp1o/Aqp1b) is trafficked to the oocyte cortex and transiently inserted into the oocyte plasma membrane during meiotic maturation, thereby mediating rapid water influx [12]. Ferré García [16] further proposed that the expression, intracellular trafficking, and post-translational regulation of Aqp1ab constitute a key molecular basis of egg hydration in marine teleosts.

Notably, in the present study, *AQP1b* expression was highest at S1 and gradually declined during maturation. This expression pattern resembles that reported in *Anguilla japonica* [38] and *Heteropneustes fossilis* [39], suggesting that AQP1b mRNA is likely accumulated and stored during the oocyte growth phase [40]. Previous studies have shown that the functional activity of AQP1b depends primarily on post-translational modification and membrane localization, rather than on simple transcriptional upregulation. Fabra et al. [12] demonstrated in sea bass (*Sparus aurata*) that membrane insertion of Aqp1b occurs during the peak of hydration, and that phosphorylation of its cytoplasmic tail serves as a key molecular switch regulating its trafficking from intracellular vesicles to the plasma membrane. Therefore, the decline in AQP1b transcript abundance in *E. coioides* may reflect maternal mRNA remodeling in preparation for ovulation and subsequent embryonic development, whereas its water channel function during final maturation is likely mediated predominantly by post-translational regulation of pre-synthesized protein. Taken together, oocyte hydration in *E. coioides* appears to result from the coordinated action of yolk proteolysis, ion redistribution, and aquaporin mobilization.

### 4.2. Temporal Regulation of Endogenous mRNAs in Oocytes

A central biological event during oocyte maturation is the post-transcriptional regulation of maternal mRNAs. Because transcriptional activity declines sharply or even becomes largely arrested after meiotic resumption, both oocyte maturation and early embryonic development rely almost entirely on the temporally controlled translational activation and selective degradation of maternal mRNAs stored during oogenesis [41,42,43]. The present study revealed a clear temporal pattern of endogenous mRNA dynamics during oocyte maturation in *E. coioides*, characterized by a transition from basal growth maintenance to metabolic remodeling and ultimately to terminal ovulatory response.

Notably, the largest number of DEGs was detected during late maturation, and these genes were significantly enriched in processes such as cell cycle, spliceosome, ribosome biogenesis, and oxidative phosphorylation, indicating that the terminal maturation stage may represent a critical window for the systematic selection and reorganization of maternal mRNAs. In teleost oocytes, the large pool of maternal mRNAs and proteins accumulated during oogenesis supports fertilization and sustains early embryonic development prior to zygotic genome activation [44]. These maternal transcripts are stored in a translationally repressed state, often with relatively short poly(A) tails. Upon meiotic resumption, RNA-binding factors including CPEB promote cytoplasmic polyadenylation of selected transcripts, such as cyclin B1, thereby activating their translation [42,45]. In this study, the enhanced nucleic acid processing, translation-related activity, and mitochondrial function observed at the final maturation stage in the present study may represent important molecular hallmarks of high-quality mature oocytes. Studies in rainbow trout (*Oncorhynchus mykiss*) and other fishes have likewise shown that the quality and composition of maternal transcripts directly influence egg quality and embryonic survival [46,47].

The trend analysis further supports this interpretation. Large numbers of genes associated with ribosomes, rRNA processing, DNA replication, and basal growth maintenance in Clusters 2 and 5 were progressively downregulated, whereas genes involved in RNA surveillance, spliceosome function, mitochondrial translation, and terminal maturation-associated responses in Clusters 1 and 6 were markedly enhanced during late maturation. These results suggest that *E. coioides* oocytes prepare for completion of GVBD, fertilization, and early development prior to zygotic genome activation through fine-scale selection and processing of maternal transcripts, together with reinforcement of mitochondrial function and ATP supply. This pattern is highly consistent with the molecular features associated with high-quality egg formation in species such as *O. mykiss* [48] and *Hippoglossus hippoglossus* [49]. Notably, ZAR1 (assigned to Cluster 5) was significantly downregulated from S1 to S3, suggesting that its decline may represent an important molecular switch associated with the transition of oocytes from the growth phase to the maturational phase [50,51]. In mammalian oocytes, ZAR1 has been identified as a critical maternal-effect gene, and loss of ZAR1/2 results in impaired meiotic maturation, abnormal maternal mRNA degradation, and failure of zygotic genome activation [52,53]. ZAR1/2 can bind maternal mRNAs and regulate the translational activation of key cell-cycle regulators, including cyclin B1 and WEE2, through 3′UTR-dependent mechanisms during oocyte maturation [53]. Our findings therefore suggest that, in marine teleosts, ZAR1 may likewise play a pivotal role in the transcriptional and post-transcriptional regulatory program underlying meiotic resumption.

### 4.3. miRNAs Participate in the Fine Post-Transcriptional Regulation of Oocyte Maturation

Accumulating evidence has shown that miRNAs in fish ovaries exhibit pronounced developmental stage specificity and play pivotal roles in folliculogenesis, oocyte maturation, ovulation, and overall reproductive success [54,55]. In the present study, miRNA-seq identified a large number of DE miRNAs, particularly let-7d-5p, miR-22a-3p, and novel-miR-20/27/118, whose target genes were significantly enriched in pathways related to ribosomal function, DNA replication, and cell cycle. Notably, the present study showed that the let-7 family member let-7d-5p targets genes associated with synaptic and vesicular transport, such as *SYNGR2* and *SRPX2*, suggesting that the let-7 family may play important regulatory roles in vesicle trafficking and cytoskeletal remodeling during oocyte maturation [55]. In mammals, members of the let-7 family have been shown to be critically involved in oocyte maturation and early embryonic development, and aberrant let-7 expression is closely associated with reduced oocyte quality [56]. Additionally, miR-22a-3p and novel-miR-20/27 were found to co-target the transcription factor E2F6. Members of the E2F family are key regulators of G1/S and G2/M transitions, and E2F6 generally functions as a transcriptional repressor involved in cell-cycle exit [57]. In *E. coioides*, miRNA-mediated inhibition of E2F6 may relieve its repressive effect on cell-cycle-related genes, thereby facilitating meiotic resumption. During meiotic resumption, spindle assembly, chromosome segregation, and polar body extrusion depend heavily on the dynamic disassembly and reorganization of the microtubule network [58,59]. The finding that novel-miR-118 targets the tubulin gene *TUBB4A* indicates that miRNAs may directly participate in cytoskeletal remodeling, a process essential for asymmetric division and polar body extrusion in oocytes [60].

Further construction of the miRNA–TF–mRNA regulatory network demonstrated that gene regulation during oocyte maturation is not a simple one-to-one relationship, but rather exhibits a pronounced hierarchical organization. For example, miR-22a-3p indirectly regulates immune- and apoptosis-related genes such as *CD44*, *SIGLEC1*, and *RIPK3* through repression of *PLEK* [61,62,63]. PLEK (pleckstrin) is a major substrate of protein kinase C (PKC) and is broadly involved in cytoskeletal reorganization and transmembrane signal transduction [64]. The identification of the miR-22a-3p–*PLEK*–*CD44*/*SIGLEC1*/*RIPK3* regulatory axis in the present study suggests the existence of a miRNA-driven immune–cell communication regulatory module during oocyte maturation, which may be associated with adaptive preparation for microenvironmental changes occurring during late maturation and after ovulation. In addition, certain core genes, such as *ITM2B* and *GPR97*, were found to be simultaneously subject to positive regulation by *PLEK* and direct repression by miR-22a-3p. Such a dual constraint mechanism may ensure that these target genes function within precise temporal windows during oocyte maturation. Likewise, novel-miR-20/27 jointly targeted and repressed *E2F6*, whereas *E2F6* positively regulated genes closely associated with the cell cycle and differentiation, including *MAEA* and *VRK1*. MAEA, a core catalytic subunit of the CTLH E3 ubiquitin ligase complex, is known to regulate cellular metabolism through non-proteolytic ubiquitination of proteins such as PKM2 and LDHA, as well as through the degradation of specific substrates including HBP1 and HMGCS1. In the context of oocyte maturation, MAEA may indirectly facilitate meiotic progression by contributing to the maintenance of intracellular metabolic homeostasis [65,66]. VRK1 is a key regulator of cell-cycle progression, and defects in VRK1 can lead to abnormal chromosome conformation and delayed cell division, ultimately impairing oocyte fertilization competence [67,68]. Similar regulatory patterns have also been reported in circRNA–miRNA–mRNA networks associated with ovarian maturation in teleosts, in which multiple differentially expressed genes were enriched in pathways such as oocyte meiosis, progesterone-mediated oocyte maturation, and cell cycle [69,70].

## 5. Conclusions

This study provides, for the first time, a systematic characterization of the temporal mRNA and miRNA regulatory landscape underlying oocyte maturation and hydration in a marine teleost producing pelagic eggs, the orange-spotted grouper (*E. coioides*). The results showed that oocyte maturation in this species is not merely an event of meiotic resumption, but rather a continuous and highly coordinated process integrating yolk proteolysis, ionic redistribution, aquaporin mobilization, maternal mRNA remodeling, enhanced mitochondrial energy metabolism, and terminal maturation responses. miRNA profiling further suggested that let-7d-5p, miR-22a-3p, and novel-miR-20/27/118 may participate in the regulation of maturation by modulating cell cycle progression, cytoskeletal remodeling, vesicular transport, and immune-related signaling pathways. In addition, the constructed miRNA–TF–mRNA regulatory network revealed a close coupling between transcriptional and post-transcriptional regulation, providing an important framework for identifying key regulatory factors involved in oocyte maturation. Overall, this study overcomes the limitations of conventional follicle-based sequencing approaches and deepens our understanding of the regulatory network governing germ cell development in marine teleosts. These findings also provide valuable molecular targets for the assessment of egg quality and the optimization of breeding strategies in marine fish. Future studies integrating in situ expression analysis, functional gene manipulation, and protein-level validation will be required to further elucidate the precise roles of key miRNAs, transcription factors, and target genes in meiotic resumption, hydration, and egg quality formation.

## Data Availability

All data are available from the corresponding author by request.

## Supplementary Materials

The following supporting information can be downloaded at: https://www.mdpi.com/article/10.3390/ani16101549/s1, Figure S1. Changes in oocyte diameter, weight, and ion content during oocyte maturation; Figure S2. Clustered heatmap (A) and PCA plot (B) of oocyte samples at different maturation stages; Figure S3. PCA plot (A) and Venn diagram (B) of miRNA-seq data from oocyte samples at different maturation stages; Figure S4. Venn diagram (A) and bar plot (B) of DE TFs across the three comparison groups of oocytes at different maturation stages; Figure S5. qRT-PCR validation of mRNA-seq and miRNA-seq; Table S1. The sequences of primers used in this study; Table S2. Changes in free amino acid levels during oocyte maturation; Table S3. Summary of mRNA-seq data quality for oocyte; Table S4. Summary of miRNA-seq data quality for oocyte; Table S5. Length distribution of miRNAs in oocytes; Table S6. Classification and annotation statistics of miRNAs in oocytes; Table S7. Summary of predicted miRNA target genes in oocytes.
