# Supplementary material for: Deciphering the miRNA–TF–mRNA Regulatory Network Underlying Oocyte Maturation in Orange-Spotted Grouper (Epinephelus coioides): Insights from Oocyte mRNA-Seq and miRNA-Seq
Source: Animals (Basel). 2026 May 19;16(10):1549. doi: 10.3390/ani16101549 (PMC13203865; doi:10.3390/ani16101549)
Supplement: Supplementary file 1 [file animals-16-01549-s001.zip › Supplementary figures.pdf]

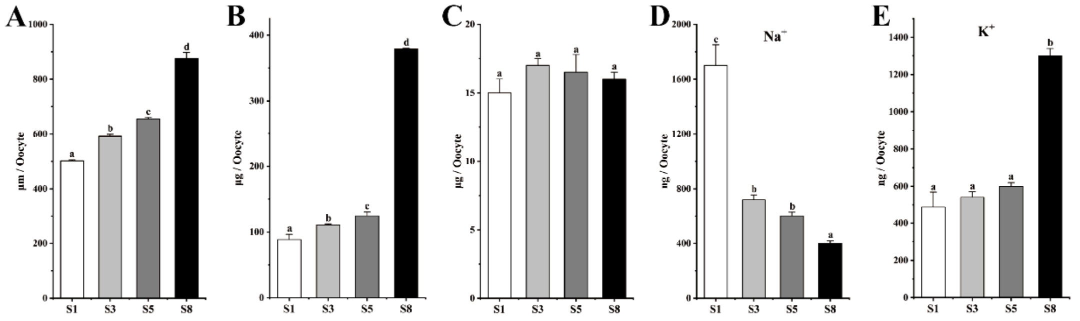

Fig. S1. Changes in oocyte diameter, weight, and ion content during oocyte maturation. (A) Oocyte diameter; (B) wet weight; (C) dry weight; (D) Na^+ concentration; (E) K^+ concentration. Different letters indicate significant differences ($P < 0.05$).

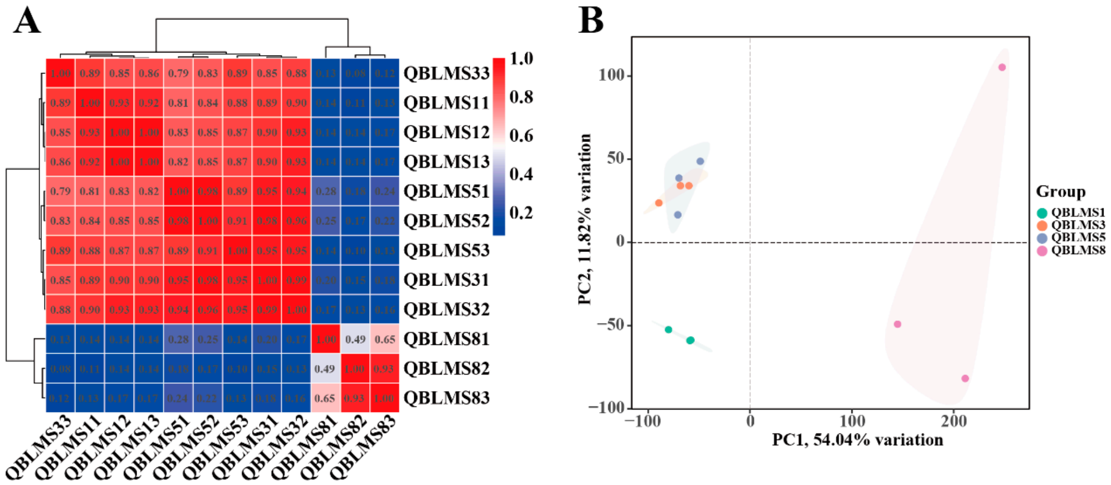

Fig. S2. Clustered heatmap (A) and PCA plot (B) of oocyte samples at different maturation stages.

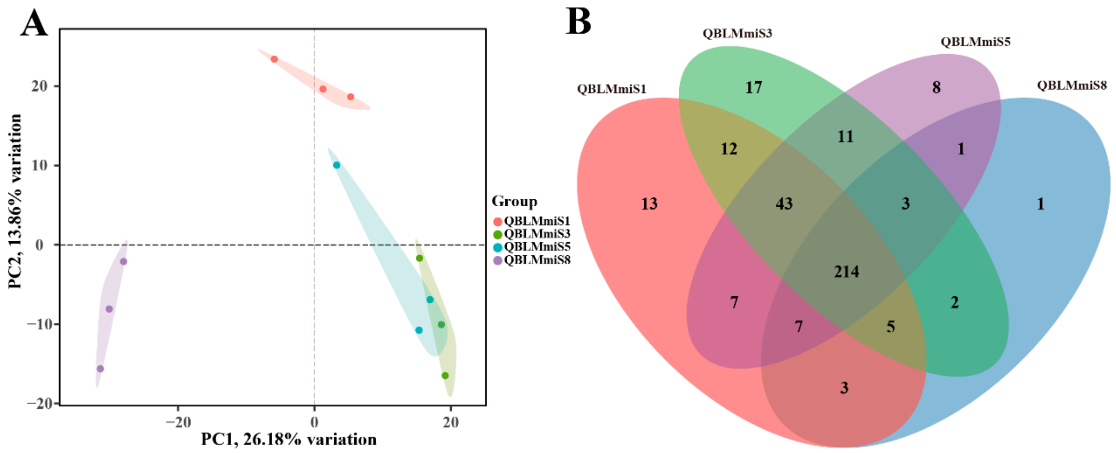

Fig. S3. PCA plot (A) and Venn diagram (B) of miRNA-seq data from oocyte samples at different maturation stages.

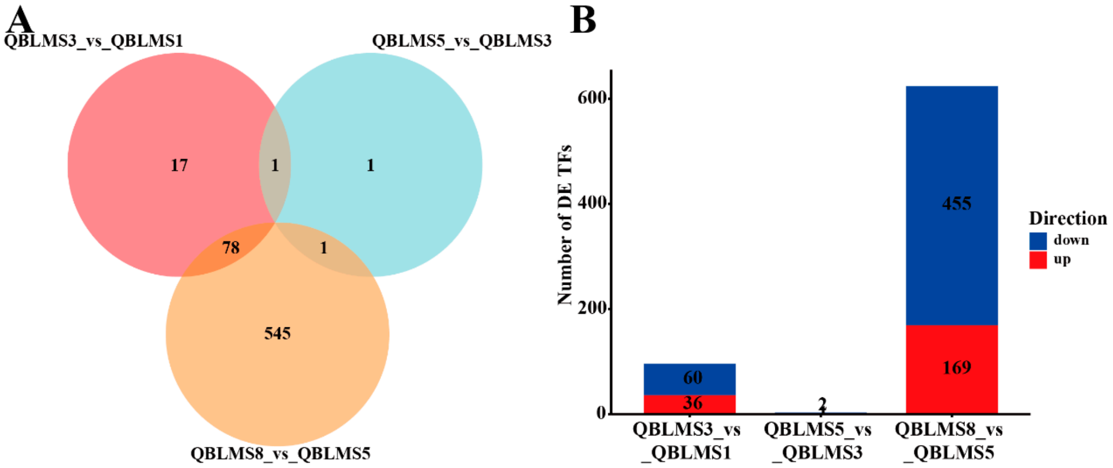

Fig. S4. Venn diagram (A) and bar plot (B) of DE TFs across the three comparison groups of oocytes at different maturation stages.

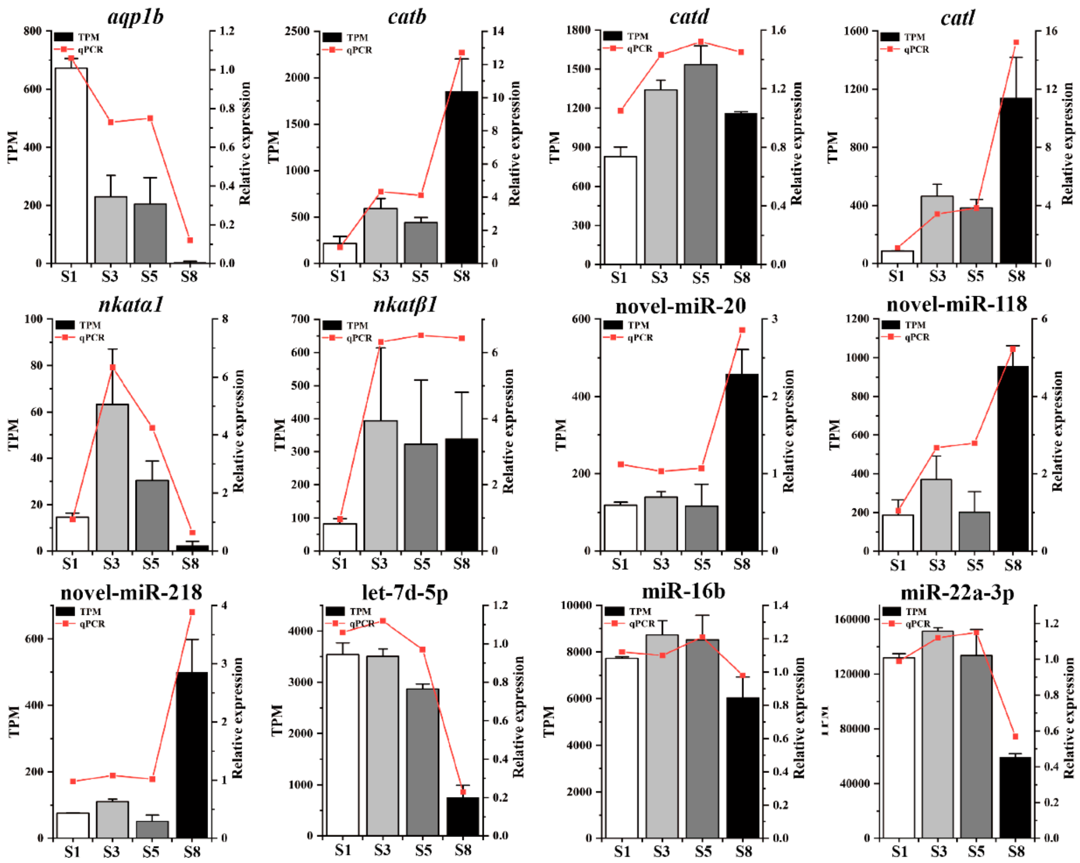

Fig. S5. qRT-PCR validation of mRNA-seq and miRNA-seq.
