# Supplementary material for: Deciphering the miRNA–TF–mRNA Regulatory Network Underlying Oocyte Maturation in Orange-Spotted Grouper (Epinephelus coioides): Insights from Oocyte mRNA-Seq and miRNA-Seq
Source: Animals (Basel). 2026 May 19;16(10):1549. doi: 10.3390/ani16101549 (PMC13203865; doi:10.3390/ani16101549)
Supplement: Supplementary file 1 [file animals-16-01549-s001.zip › Supplementary tables.pdf]

Table S1. The sequences of primers used in this study.

Gene Name		Primer (5'-3')
aqp1b	-F	CCTACCCTGACCAGGAGATAAA
	-R	GCACCGCTAATGTGACCTATG
catb	-F	TCCCACTCTGAAGGAGATCA
	-R	CTTTGCCATTGCTGTGGATAC
catd	-F	AGTCAGGACACATGCACAAT
	-R	GTCAAACTTGGCTGCGATAAAG
catl	-F	GGCCATGAGTCTTTCCAGTT
	-R	CTGGAAGCCATAACCAACGA
nkatα1	-F	CCTCTTCATCATCGCCAACA
	-R	CGCTCTCAGCTGCTTCATAA
nkatβ1	-F	TACTTTGGCATGGGTGAAGG
	-R	CCTGTGGTGAGGTTGGTAAA
β-actin	-F	ACCATCGGCAATGAGAGGTT
	-R	ACATCTGCTGGAAGGTGGAC
U6	-F	GCTTCGGCAGCACATATACTAAAAT
	-R	CGCTTCACGAATTTGCGTGTCAT
novel-miR-20		GAATCCACCAGCACAGAAGAA
novel-miR-118		CCGAGGCGTTCCCAGGCCAGCC
novel-miR-218		CGTCCTCTGAGCTGGCAGGGA
let-7d-5p		AACCATACAACCAACTACCTCA
miR-16b		CTCCAATATTTACGTGCTGCTA
miR-22a-3p		ACAGTTCTTCAGCTGGCAGCTT

Table S2. Changes in free amino acid levels during oocyte maturation.

Compounds	QBLMS1		QBLMS3		QBLMS8	
	Content ng/oocyte	Proportion （%）	Content ng/oocyte	Proportion （%）	Content ng/oocyte	Proportion （%）
L-Phenylalanine	10.88 ± 0.54	1.05 ±0.03	48.18 ± 2.24	2.27 ± 0.03	137.63 ± 9.24	1.67 ± 0.03
L-Serine	12.36 ± 1.02	1.20 ± 0.01	41.84 ± 4.20	1.97 ± 0.22	311.06 ± 7.88	3.77 ± 0.05
L-Threonine	7.70 ± 0.83	0.75 ± 0.00	51.09 ± 3.83	2.41 ± 0.10	374.31 ± 4.20	4.53 ± 0.01
L-Lysine	114.27 ± 8.40	11.08 ± 0.52	223.33 ± 7.68	10.51 ± 0.82	605.50 ± 3.90	7.34 ± 0.01
L-Histidine	17.98 ± 1.11	1.74 ± 0.03	32.18 ± 3.31	1.51 ± 0.03	118.00 ± 1.77	1.43 ± 0.00
L-Isoleucine	10.13 ± 2.30	0.98 ± 0.01	63.63 ± 3.40	3.00 ± 0.11	366.22 ± 5.21	4.44 ± 0.04
L-Glutamic acid	43.77 ± 0.58	4.24 ± 0.24	105.86 ± 8.22	4.98 ± 0.60	926.83 ± 6.22	11.23 ± 0.21
L-Ornithine	10.46 ± 1.23	1.01 ± 0.02	64.72 ± 7.62	3.05 ± 0.03	70.47 ± 0.84	0.85 ± 0.06
L-Leucine	28.91 ± 3.20	2.80 ± 0.03	138.27 ± 9.30	6.51 ± 0.39	318.16 ± 6.24	3.85 ± 0.11
L-Glutamine	215.25 ± 9.70	20.87 ± 0.12	306.05 ± 5.23	14.41 ± 1.11	1641.90 ± 30.91	19.89 ± 1.23
L-Methionine	5.27 ± 0.22	0.51 ± 0.06	19.34 ± 3.10	0.91 ± 0.01	147.74 ± 2.99	1.79 ± 0.02
L-Alanine	27.80 ± 1.53	2.69 ± 0.11	124.31 ± 4.01	5.85 ± 0.6	434.23 ± 6.72	5.26 ± 0.18
N-Acetylneuraminic Acid	0.08 ±0.01	0.01 ± 0.00	0.41 ± 0.02	0.02 ± 0.00	0.92 ± 0.06	0.01 ± 0.00
L-Aspartate	4.98 ± 0.07	0.48 ± 0.03	10.23 ± 1.20	0.48 ± 0.02	31.83 ± 5.21	0.39 ± 0.01
L-Proline	12.94 ± 1.22	1.25 ± 0.21	70.09 ± 6.00	3.30 ± 0.04	281.84 ± 9.00	3.41 ± 0.03
L-Asparagine Anhydrous	10.78 ± 0.83	1.04 ± 0..04	35.44 ± 5.30	1.67 ± 0.12	187.95 ± 6.83	2.28 ± 0.02
Trans-4-Hydroxy-L-Proline	2.80 ± 0.55	0.27 ± 0.03	3.56 ± 0.42	0.17 ± 0.01	7.52 ± 0.55	0.09 ± 0.00
S-(5-Adenosyl)-L-Homocysteine	0.34 ± 0.03	0.03 ± 0.00	0.46 ± 0.06	0.02 ±0.00	3.33 ± 0.20	0.04 ± 0.00
L-Arginine	294.70 ± 7.54	28.57 ± 0.22	320.27 ± 2.30	15.08 ± 1.20	704.60 ± 6.12	8.54 ± 0.22
L-Tyrosine	13.48 ± 0.69	1.31 ±0.01	43.79 ± 2.01	2.06 ± 0.01	170.69 ± 3.33	2.07 ± 0.01
Ethanolamine	19.50 ± 2.02	1.89 ± 0.07	31.40 ± 4.33	1.48 ±0.02	11.58 ± 2.01	0.14 ± 0.00
α-Aminoadipic acid	2.49 ± 0.09	0.24 ± 0.02	10.30 ± 0.59	0.48 ± 0.01	4.86 ± 0.02	0.06 ± 0.00
1-Methylhistidine	1.24 ± 0.04	0.12 ± 0.01	0.64 ± 0.03	0.03 ± 0.00	1.42 ± 0.06	0.02 ± 0.00
Creatine	52.54 ± 1.78	5.09 ± 0.09	74.84 ± 3.55	3.52 ± 0.03	99.09 ± 3.76	1.20 ± 0.02
2-Aminoethanesulfonic Acid	30.09 ± 3.20	2.92 ± 0.05	17.07 ± 1.00	0.80 ± 0.02	37.15 ± 2.01	0.45 ± 0.02
Glycine	9.65 ± 0.88	0.94 ± 0.02	20.16 ± 0.98	0.95 ± 0.01	135.33 ± 1.09	1.64 ± 0.03
Trimethylamine N-Oxide	5.76 ± 0.57	0.56 ± 0.01	3.04 ± 0.06	0.14 ± 0.00	1.62 ± 0.01	0.02 ± 0.00
L-Tryptophan	7.17 ± 1.51	0.70 ± 0.02	29.26 ± 1.11	1.38 ± 0.06	61.82 ± 1.59	0.75 ± 0.04
L-Valine	12.39 ± 1.25	1.20 ± 0.06	93.96 ± 4.23	4.42 ± 0.29	311.47 ± 6.20	3.77 ± 0.06
N6-Acetyl-L-Lysine	0.02 ± 0.00	0.00 ± 0.00	0.07 ± 0.01	0.00 ± 0.00	0.92 ± 0.03	0.01 ± 0.00
glycylphenylalanine	0.22 ± 0.00	0.02 ± 0.00	0.58 ± 0.01	0.03 ±0.00	0.48 ± 0.02	0.01 ± 0.00
Methionine Sulfoxide	0.09 ± 0.00	0.01 ± 0.00	0.16 ± 0.00	0.01 ± 0.00	0.32 ± 0.01	0.00 ± 0.00
L-Cystathionine	0.00 ± 0.00	0.00 ± 0.00	2.05 ± 0.02	0.10 ± 0.00	2.97 ± 0.22	0.04 ± 0.00

N-Glycyl-L-Leucine	1.08 ± 0.06	0.10 ± 0.01	2.55 ± 0.06	0.12 ±0.02	1.68 ± 0.02	0.02 ±0.00
O-Phospho-L-Serine	15.21 ± 1.09	1.47 ± 0.11	12.84 ± 1.84	0.60 ± 0.01	29.97 ± 2.09	0.36 ± 0.02
L-Carnosine	2.04 ± 0.02	0.20 ± 0.02	57.96 ± 6.88	2.73 ± 0.10	0.97 ± 0.05	0.01 ± 0.00
L-Citrulline	1.48 ± 0.03	0.14 ± 0.01	0.83 ± 0.06	0.04 ± 0.00	2.03 ± 0.03	0.02 ± 0.00
Glycyl-L-Proline	0.30 ± 0.01	0.03 ± 0.00	0.24 ± 0.02	0.01 ± 0.00	0.88 ± 0.05	0.01 ± 0.00
3-N-Methyl-L-Histidine	0.35 ± 0.00	0.03 ± 0.00	1.67 ± 0.01	0.08 ± 0.00	2.18 ± 0.11	0.03 ± 0.00
argininosuccinic acid	0.00 ± 0.00	0.00 ± 0.00	0.00 ± 0.00	0.00 ± 0.00	496.42 ± 9.76	6.01 ± 0.03
γ-Glutamate-Cysteine	25.03 ± 2.09	2.43 ± 0.02	46.49 ± 8.01	2.19 ± 0.09	11.05 ± 1.01	0.13 ± 0.01
D-Alanyl-D-Alanine	0.00 ± 0.00	0.00 ± 0.00	0.34 ± 0.02	0.02 ± 0.00	0.17 ± 0.03	0.00 ± 0.00
2-Aminobutyric acid	0.00 ± 0.00	0.00 ± 0.00	1.45 ± 0.01	0.07 ± 0.01	5.51 ± 0.50	0.07 ± 0.00
Phosphorylethanolamine	0.00 ± 0.00	0.00 ± 0.00	0.00 ± 0.00	0.00 ± 0.00	86.88 ± 6.29	1.05 ± 0.02
Glutathione Oxidized	0.00 ± 0.00	0.00 ± 0.00	0.00 ± 0.00	0.00 ± 0.00	7.10 ± 0.80	0.09 ± 0.00
L-Cysteine	0.00 ± 0.00	0.00 ± 0.00	8.60 ± 0.11	0.40 ± 0.02	35.87 ± 2.20	0.43 ± 0.03
Beta-Alanine	0.00 ± 0.00	0.00 ± 0.00	0.74 ± 0.05	0.03 ± 0.00	3.14 ± 0.60	0.04 ± 0.00
Nα-Acetyl-L-Arginine	0.00 ± 0.00	0.00 ± 0.00	0.00 ± 0.00	0.00 ± 0.00	0.03 ± 0.00	0.00 ± 0.00
L-Cystine	0.00 ± 0.00	0.00 ± 0.00	1.78 ± 0.10	0.08 ± 0.01	4.43 ± 0.03	0.05 ± 0.00
Homo-L-arginine	0.00 ± 0.00	0.00 ± 0.00	0.44 ± 0.02	0.02 ± 0.00	0.47 ± 0.02	0.01 ± 0.00
D-Homocysteine	0.00 ± 0.00	0.00 ± 0.00	1.73 ± 0.06	0.08 ± 0.01	0.66 ± 0.02	0.01 ± 0.00
Urea	0.00 ± 0.00	0.00 ± 0.00	0.00 ± 0.00	0.00 ± 0.00	25.43 ± 3.02	0.31 ± 0.01
N,N-Dimethylglycine	0.00 ± 0.00	0.00 ± 0.00	0.00 ± 0.00	0.00 ± 0.00	0.06 ± 0.00	0.00 ± 0.00
L-Pipecolic Acid	0.00 ± 0.00	0.00 ± 0.00	0.00 ± 0.00	0.00 ± 0.00	1.43 ± 0.01	0.02 ± 0.00
L-Homocitrulline	0.00 ± 0.00	0.00 ± 0.00	0.00 ± 0.00	0.00 ± 0.00	0.31 ± 0.02	0.00 ± 0.00
L-α-Aspartyl-L-phenylalanine	0.00 ± 0.00	0.00 ± 0.00	0.00 ± 0.00	0.00 ± 0.00	0.02 ± 0.00	0.00 ± 0.00
Succinic Acid	0.00 ± 0.00	0.00 ± 0.00	0.00 ± 0.00	0.00 ± 0.00	16.44 ± 1.11	0.20 ± 0.00
L-Tryptophyl-L-glutamic acid	0.00 ± 0.00	0.00 ± 0.00	0.00 ± 0.00	0.00 ± 0.00	8.58 ± 0.59	0.10 ± 0.00
4-Acetamidobutyric Acid	0.00 ± 0.00	0.00 ± 0.00	0.00 ± 0.00	0.00 ± 0.00	0.08 ± 0.01	0.00 ± 0.00
L-Theanine	0.00 ± 0.00	0.00 ± 0.00	0.00 ± 0.00	0.00 ± 0.00	0.08 ± 0.01	0.00 ± 0.00
N8-Acetylspermidine	0.00 ± 0.00	0.00 ± 0.00	0.00 ± 0.00	0.00 ± 0.00	3.06 ± 0.06	0.04 ± 0.00
L-tyrosine methyl ester	0.00 ± 0.00	0.00 ± 0.00	0.00 ± 0.00	0.00 ± 0.00	0.03 ± 0.00	0.00 ± 0.00
Total	1031.52 ± 16.33	100.00	2124.26 ± 30.21	100.00	8254.68 ±12.74	100.00

Table S3. Summary of mRNA-seq data quality for oocyte.

Sample	Raw Reads	Raw Data (bp)	Clean Reads	Clean Data (bp)	Q20 (%)	Q30 (%)	GC (%)	Unique Mapped (%)	Multiple_ Mapped (%)	Total Mapped (%)
QBLMS11	36,994,602	5,549,190,300	36,620,534	5,528,934,720	98.45%	95.19%	47.57%	88.38%	4.32%	92.70%
QBLMS12	43,408,330	6,511,249,500	42,750,496	6,482,582,961	98.45%	95.22%	46.97%	88.43%	3.36%	91.79%
QBLMS13	43,180,534	6,477,080,100	42,574,342	6,453,113,995	98.46%	95.25%	47.12%	88.02%	3.60%	91.62%
QBLMS31	43,318,524	6,497,778,600	42,881,406	6,477,318,322	98.54%	95.43%	45.94%	89.27%	2.25%	91.52%
QBLMS32	41,764,718	6,264,707,700	41,397,830	6,240,357,257	98.55%	95.49%	45.79%	89.10%	2.55%	91.65%
QBLMS33	42,541,621	6,381,243,150	42,139,618	6,358,837,790	98.55%	95.46%	45.87%	89.19%	2.40%	91.59%
QBLMS51	49,134,138	7,370,120,700	48,295,748	7,337,185,459	98.49%	95.35%	46.38%	88.18%	2.80%	90.98%
QBLMS52	41,646,916	6,247,037,400	41,243,520	6,222,651,083	98.44%	95.22%	45.30%	88.11%	3.05%	91.16%
QBLMS53	36,332,230	5,449,834,500	35,460,086	5,422,646,013	98.33%	94.90%	46.52%	88.00%	3.50%	91.50%
QBLMS81	36,120,908	5,418,136,200	35,639,652	5,398,351,523	98.44%	95.14%	44.39%	89.25%	1.51%	90.76%
QBLMS82	47,900,286	7,185,042,900	47,216,446	7,158,753,884	98.48%	95.25%	43.89%	89.44%	1.57%	91.01%
QBLMS83	46,670,172	7,000,525,800	45,878,492	6,971,176,617	98.51%	95.38%	43.53%	89.61%	1.57%	91.18%

Table S4. Summary of miRNA-seq data quality for oocyte.

Sample	Raw reads	High quality	3’adapter null	Insert null	PolyA	Clean reads
QBLMmiS11	16,263,436	15,859,773	618,219	147,996	547	15,026,878
QBLMmiS12	14,152,341	13,948,532	197,639	180,022	962	12,518,736
QBLMmiS13	14,971,529	14,810,087	297,375	176,521	1,131	13,178,284
QBLMmiS31	12,026,664	11,886,387	258,460	888,917	879	10,321,836
QBLMmiS32	16,690,983	16,281,898	281,237	484,157	1,647	13,956,282
QBLMmiS33	14,358,824	14,084,143	269,849	686,537	1,263	12,139,059
QBLMmiS51	12,103,095	12,034,055	254,647	136,224	1,212	11,078,023
QBLMmiS52	9,508,277	9,453,910	229,213	111,323	818	8,448,798
QBLMmiS53	11,034,264	10,817,344	351,819	108,560	567	9,703,586
QBLMmiS81	17,203,366	16,762,765	386,609	156,512	353	15,577,596
QBLMmiS82	14,186,008	13,895,503	231,634	639,92	227	12,227,615
QBLMmiS83	12,015,099	11,904,189	191,671	813,78	216	11,044,543

Table S5. Length distribution of miRNAs in oocytes.

length (nt)	18	19	20	21	22	23	24	25	26	27	28	29	30	31	32	33	34	35
proportion (%)	8.69	9.92	10.19	11.19	12.80	13.22	8.82	6.42	4.82	4.14	3.08	1.96	1.11	1.17	0.80	0.88	0.53	0.25

Table S6. Classification and annotation statistics of miRNAs in oocytes.

Sample	Total	rRNA	snRNA	snoRNA	tRNA	Known miRNA	Novel miRNA	Transcriptome	Unannotated
QBLMmiS11	15,026,878	3,163,158	9,016	12,022	694,242	1,247,231	10,519	830,986	9,059,705
QBLMmiS12	12,518,736	2,587,623	5,007	20,030	525,787	1,269,400	15,022	649,722	7,447,396
QBLMmiS13	13,178,284	2,592,168	5,271	22,403	690,542	1,344,185	17,132	611,472	7,893,792
QBLMmiS31	10,321,836	2,781,735	2,064	7,225	527,446	1,102,372	7,225	400,487	5,492,249
QBLMmiS32	13,956,282	3,821,230	2,791	6,978	676,880	1,659,402	11,165	535,921	7,241,915
QBLMmiS33	12,139,059	3,297,575	2,428	7,283	604,525	1,369,893	9,104	468,568	6,379,076
QBLMmiS51	11,078,023	2,601,120	2,216	8,862	499,619	1,200,858	9,970	440,905	6,315,581
QBLMmiS52	8,448,798	1,928,861	1,690	5,914	412,301	751,943	5,914	381,886	4,960,289
QBLMmiS53	9,703,586	2,842,180	4,852	21,348	475,476	873,323	13,585	393,966	5,079,827
QBLMmiS81	15,577,596	10,175,286	6,231	7,789	130,852	721,243	4,673	451,750	4,078,215
QBLMmiS82	12,227,615	8,966,510	4,891	6,114	217,652	338,705	2,446	207,869	2,483,429
QBLMmiS83	11,044,543	7,553,363	3,313	5,522	162,355	482,647	3,313	300,412	2,532,514

Table S7. Summary of predicted miRNA target genes in oocytes.

Sample name	miRNA number	Target gene number	Target site number
Total	344	14,630	65,520
QBLMmiS11	226	13,178	41,765
QBLMmiS12	268	13,842	51,081
QBLMmiS13	269	13,765	49,676
QBLMmiS31	240	13,657	47,470
QBLMmiS32	275	14,152	55,673
QBLMmiS33	246	13,468	46,646
QBLMmiS51	257	13,831	51,296
QBLMmiS52	233	13,497	45,004
QBLMmiS53	239	13,420	46,040
QBLMmiS81	198	12,695	37,518
QBLMmiS82	174	11,986	33,141
QBLMmiS83	181	12,349	34,280
